# Plasma apolipoprotein E and monocyte chemoattractant protein-1 levels in young people with HIV and ischemic stroke in Lusaka, Zambia

**DOI:** 10.3389/fstro.2025.1595809

**Published:** 2025-11-20

**Authors:** Stanley Zimba, Owen Ngalamika, Emmanuel Mukambo, Mike Chisha, Violet Kayamba, Lloyd Mulenga, Omar Siddiqi, Deanna Saylor, Owen A. Ross, Masharip Atadzhanov

**Affiliations:** 1 Department of Internal Medicine, University Teaching Hospital, Lusaka, Zambia; 2 Department of Internal Medicine, University of Zambia School of Medicine, Lusaka, Zambia; 3 Directorate of Infectious Diseases, Ministry of Health, Lusaka, Zambia; 4 Department of Neurology, Beth Israel Deaconess Medical Center, Harvard Medical School, Boston, MA, United States; 5 Department of Neurology, University of North Carolina School of Medicine, Chapel Hill, NC, United States; 6 Department of Neuroscience, Mayo Clinic College of Medicine, Jacksonville, FL, United States

**Keywords:** apolipoprotein E, monocyte chemoattractant protein 1, sub-Saharan Africa, young onset stroke, HIV infection

## Abstract

**Background:**

Apolipoprotein E (ApoE) and monocyte chemoattractant protein-1 (MCP-1) are inflammatory markers associated with premature atherosclerosis, which leads to increased cardiovascular disease risk among people with HIV (PWH). We aimed to evaluate the association between the plasma levels of these inflammatory markers and ischemic stroke in young PWH.

**Methods:**

We conducted a prospective case-control study at the University Teaching Hospital in Lusaka, Zambia, between March 2022 and October 2024, comparing young PWH with non-cardioembolic ischemic stroke (cases) to age- and sex-matched PWH without a history of stroke (controls). Standardized data collection instruments were used to collect information on other known risk factors for stroke, including demographic, clinical, laboratory, and imaging parameters. ELISA was done to measure ApoE and MCP-1 levels in the plasma of individuals in both the case and control groups.

**Results:**

We analyzed results for 50 cases and 50 controls. Compared to controls, cases were more likely to have (1) traditional stroke risk factors such as hypertension (42 vs. 2%, *p* = 0.001); (2) more poorly controlled HIV, including lower CD4 counts [259 (165–520) cells/μl vs. 452 (380–553) cells/μl, *p* = 0.030)] and higher viral loads [0 (0–4,217) copies/ml vs. 0 (0–1,578) copies/ml, *p* = 0.007]; (3) markers of atherosclerotic disease, including increased pulse wave velocity (PWV) [10.89 (9.99–12.15) m/s vs. 9.01 (7.989.67) m/s, *p* < 0.001] and carotid intima-media thickness (cIMT) [0.79 (0.70–0.99) mm vs. 0.63 (0.57–0.67) mm, *p* < 0.001]. Cases had lower plasma ApoE levels [1.20 (0.78–1.41) ng/ml vs. 1.55 (1.23–1.81) ng/ml, *p* = 0.001], but not statistically different MCP-1 plasma levels [622 (417–886) pg/ml vs. 594 (394–1,024) pg/ml, *p* = 0.772] compared to controls. Lower ApoE levels (aOR 0.13, 95% CI 0.03–0.68, *p* = 0.015), abnormal cIMT ≥0.70 mm (aOR 2.72, 95% CI 1.08–6.85, *p* = 0.033), and alcohol use (aOR 1,078, 95% CI 4–267,933, *p* = 0.013) were independently associated with ischemic stroke in multivariable analysis.

**Conclusion:**

The results suggest that lower plasma ApoE levels are independently associated with non-cardioembolic ischemic stroke in young PWH. Additional studies with larger sample sizes are needed to further explore the contribution of these inflammatory markers in young-onset HIV-associated stroke.

## 1 Introduction

Atherosclerosis has been reported to occur at higher rates and at younger ages in people with HIV (PWH), and could be associated with increased risk of cardiovascular and cerebrovascular diseases (Zimba et al., 2021; Hsue et al., 2012; Piconi and Clerici, 2013; Triant, 2012; Piconi et al., 2013; Albuquerque et al., 2013). For instance, HIV-associated stroke tends to occur at younger ages (< 50 years of age) compared to people without HIV (PWoH; Zimba et al., 2021). The underlying aetiological mechanisms for increased risk of stroke among PWH remain unclear (Hsue et al., 2012; Piconi and Clerici, 2013). There is evidence that inflammatory markers are elevated in PWH in comparison with PWoH, (Piconi and Clerici, 2013; Triant, 2012) potentially leading to premature atherosclerosis and young-onset stroke. Although PWH have a high prevalence of traditional risk factors, they also have other factors that may induce premature atherosclerosis and lead to young-onset stroke such as immunosuppression, chronic inflammation, HIV-induced foam cell transformation, and cumulative exposure to antiretroviral therapy (ART) medications which cause mitochondrial and metabolic dysfunction (Piconi et al., 2013; Albuquerque et al., 2013).

Several inflammatory pathways are implicated in the development of atherosclerosis, with some, such as mutations of monocyte chemoattractant protein-1 (*MCP-1*) and apolipoprotein E (*APOE*) genes, due to genetic predispositions (Roy et al., 2009; Ibáñez et al., 2014). Circulating monocytes are attracted to the subendothelial space mainly by MCP-1, where they commence phagocytosis of modified lipoproteins and become fat-laden foam cells (Charo and Taubman, 2004; Gu et al., 1997). Several studies have implicated an MCP-1 gene mutation with higher protein expression, making them more susceptible to developing premature atherosclerosis and potentially resulting in young-onset stroke (Rovin et al., 1999; Alonso-Villaverde et al., 2004). However, little is known about the influence of the presence of HIV on this gene mutation and the impact that might have on atherosclerosis and increasing young-onset stroke risk.

ApoE has a protective role in cardiovascular diseases. It mediates high-affinity binding of ApoE-containing lipoproteins to low-density lipoprotein receptors (LDL-R) and LDL-R-related protein-1, facilitating the clearance of triglyceride-rich lipoproteins from circulation and conferring protection from atherosclerosis (Sofat et al., 2016). Very low circulating levels of ApoE are associated with premature atherosclerosis (Schaefer et al., 1986). A previous study in Zambia found an increased risk of haemorrhagic stroke in individuals with the *ApoE* ε*2*ε*4* genotype, but correlations with plasma ApoE levels were not investigated, and no evaluations on the association with HIV or young-onset stroke were made (Atadzhanov et al., 2013).

The main aim of this exploratory study was to investigate plasma MCP-1 and ApoE concentrations as risk factors for HIV-associated stroke by comparing young PWH with and without ischemic stroke. We prioritized these inflammatory markers partly because of our previous ApoE gene polymorphism findings, but also due to inadequate funding to evaluate other inflammatory markers. We used the Trial of ORG 10172 in Acute Stroke Treatment (TOAST) classification (Goldstein et al., 2001) to select only individuals with non-cardioembolic ischemic strokes in order to select for individuals with a higher likelihood of having atherosclerosis. We hypothesized that non-cardioembolic ischemic stroke in young PWH is associated with premature atherosclerosis which, in turn, is associated with low ApoE and high MCP-1 plasma concentrations.

## 2 Methods

### 2.1 Study setting

The study was conducted at the University Teaching Hospital (UTH) in Lusaka, Zambia, which is the largest tertiary care center in Zambia, a country of approximately 22 million people (Mulenga, 2024). Available neurodiagnostic assessments at UTH include magnetic resonance imaging (MRI), computed tomography (CT), and electroencephalography. The hospital has a neurology division with a functional stroke unit and serves as the national referral hospital. Hospital admission, physician consultations, and medications stocked in the hospital pharmacy are free of charge for all patients, but individuals may pay out of pocket for any other medications and all investigations if they do not have health insurance. As such, incomplete workups are common because of financial limitations, unavailability of laboratory reagents, and/or non-functioning neuroimaging. In addition, investigations that would not significantly alter patient management are usually not undertaken. For example, vessel imaging is not obtained as part of routine stroke care because dual antiplatelet therapy is not widely available due to the expense, and there are currently no vascular surgeons or interventional radiologists in Zambia.

### 2.2 Study design and participants

We conducted a prospective matched case-control study of young PWH with and without ischemic stroke from March 2022 to October 2024. Cases were PWH aged between 18 and 49 years with imaging-confirmed ischemic stroke presenting within 2 weeks of symptom onset and where the stroke was of non-cardioembolic etiology using the TOAST criteria. Cases were recruited from the UTH inpatient neurology service and neurology clinics. An age- (3+ years), sex-, and race-matched control was selected for each case. Controls were confirmed to be stroke-free after a neurological assessment and had no history of any other neurological disorder. Control participants were recruited while accessing routine outpatient HIV care services at UTH. Patients with sickle cell disease, cardiac diseases, meningitis, or any other central nervous system space-occupying lesion were excluded from the study. Similarly, PWoH with stroke were excluded on the basis of previous findings that they tend to be older than PWH with ischemic stroke, and young-onset stroke in PWoH is often caused by non-traditional cardiovascular risk factors (Zimba et al., 2021; Coll et al., 2007; Triant et al., 2007).

### 2.3 Study procedures

Upon obtaining informed consent, we collected sociodemographic and clinical information. Routine stroke workups, including CT and MRI scans, lipid panel, electrocardiogram, and echocardiogram, were also recorded if obtained as part of routine clinical care. All participants were assessed and examined by a neurologist. Stroke severity was graded according to the National Institutes of Health Stroke Scale (NIHSS) and the modified Rankin scale (mRS; Lyden et al., 1994; Scale, 2008). A blood sample was collected and separated into plasma which was used to determine CD4 count and HIV viral load as well as for MCP-1 and ApoE quantification.

### 2.4 Comorbid conditions case definitions

Hypertension was defined per World Health Organization (WHO) guidelines as current use of antihypertensive medication, history of being diagnosed as hypertensive by a doctor prior to stroke, documented blood pressure (BP) of greater than or equal to 140 mmHg systolic or 90 mmHg diastolic before the stroke or persisting more than a week after the acute event or evidence of left ventricular hypertrophy on electrocardiogram (ECG) or echocardiogram (Whitworth, 2003). Atrial fibrillation was defined by self-reported history, ECG, or echocardiogram. Diabetes mellitus was diagnosed if a participant was taking antidiabetic drugs prior to stroke; if a doctor had diagnosed type I or type II diabetes before stroke; if a patient had a documented hemoglobin A1c ≥6.5% or non-fasting blood glucose ≥11.1 mmol/L, or if a patient had a fasting blood glucose ≥7.0 mmol/L after the acute phase of stroke (Alberti and Zimmet, 1998). Hyperlipidaemia was defined by self-report, statin use, or standard laboratory cutoffs including low-density lipoprotein (LDL) greater than 3.36 mmol/L, high-density lipoprotein (HDL) less than 1.29 mmol/L, total cholesterol more than 5.17 mmol/L, or triglycerides greater than 3.88 mmol/L (Nelson, 2012). Cigarette smoking was classified by self-report as active smoker (current or former smoker for less than 1 year), passive smoker (household member or coworker who regularly smoked in his/her presence for more than 1 year during the last 10 years), or non-smoker, and alcohol use was defined by self-reported alcohol intake ≥1 drink per week or former alcohol intake that stopped for less than 1 year.

### 2.5 Carotid intima-media thickness and pulse wave velocity

Carotid Doppler studies were performed using a Mindray DC-40 linear probe 8–13 MHz high-resolution B-mode ultrasound and using previously described methods (Touboul et al., 2012). A carotid intima-media thickness (cIMT) ≥0.70 mm was considered to be pathological and suggestive of atherosclerosis (Lorenz et al., 2007). Large artery stiffness was indexed from aortic pulse wave velocity (PWV) using applanation tonometry, and aortic PWV was measured using previously described methods (Norton et al., 2012). A PWV above the threshold of 10.00 m/s was considered as suggestive of increased large artery stiffness (Kim and Kim, 2019). The cIMT and PWV examinations were performed by a single operator (MC),^1^ a radiologist who had undergone dedicated training in the relevant techniques. The operator was blinded to the category of the patient with regards to case or control.

### 2.6 Stroke classification

All brain imaging results were interpreted by a radiologist (MC), and stroke classification was done independently by two study investigators (SZ and MA). Stroke classification was adjudicated by a third neurologist (DS) when there was disagreement between the first two reviewers. Ischemic stroke cases were further classified using the TOAST classification: large artery atherosclerosis, cardio-embolism, small vessel occlusion (lacuna), determined etiology (vasculitis, hypercoagulability), and undetermined cause (Goldstein et al., 2001). All etiologies were included except those classified as due to cardio-embolism.

### 2.7 ELISA assays

A commercial Human ELISA ApoE kit (Biomatik, Ontario) for *in vitro* quantitative measurement in human serum was used to determine plasma ApoE levels. The assay was performed according to the manufacturer's instructions. A human MCP-1 ELISA kit (Biomatik, Ontario) was used to quantify MCP-1 levels in plasma according to the manufacturer's instructions. Serially diluted standards were incorporated in both assays in order to determine the absolute concentration of the analytes using a standard curve.

### 2.8 Sample size

This was a pilot study with limited previous studies to help with sample size calculations. Based on our prior clinical registry (Zimba et al., 2021), we estimated we could recruit 50 PWH with non-cardioembolic ischemic stroke during a 1-year recruitment period and cases were matched 1:1 with controls. Although the sample size was small, we were confident that the findings from this study would provide foundational data for future research.

### 2.9 Statistical analysis

All data were entered into a secure REDCap database hosted by the Zambian Ministry of Health Infectious Diseases Directorate and analyzed using SPSS version 27 (IBM, New York; Harris et al., 2019, 2009). Demographic and clinical characteristics were summarized using descriptive statistics. Chi-square tests were used to determine the association between categorical predictor variables and ischemic stroke. Since all the continuous variables were not normally distributed, the non-parametric Mann–Whitney *U*-test was used to determine association between ischemic stroke and continuous variables. *p* Values of less than or equal to 0.002 were taken as statistically significant in order to correct for increased type 1 error due to multiple comparisons.

A multivariable conditional logistic regression model was used to adjust for confounders and to identify factors independently associated with young-onset HIV-associated non-cardioembolic ischemic stroke. Variables that reached statistical significance in bivariable models and those associated with stroke using biological plausibility were included in the multivariate logistic regression model. Different models were run, and we selected the best predictive model based on one with the largest area under the receiver-operating characteristic (ROC) curve, lowest Akaike information criterion and Bayesian information criterion, and the highest likelihood ratio. We checked fitness of the model using Hosmer–Lemeshow goodness of fit test and confirmed that there was no multicollinearity using variance inflation factor values. *p* Values of less than 0.05 were taken as statistically significant.

### 2.10 Ethical approval

Ethical approval was obtained from the University of Zambia Biomedical Research Ethics Committee (No. 1945-2021) and the Zambia National Health Research Authority (Ref: NHREB00008/30/09/2021), and permission was obtained from UTH management to conduct the study. Written informed consent was obtained from each study participant. In the event that the participant had altered mental status, informed consent was obtained from the participant's designated surrogate decision-maker.

## 3 Results

### 3.1 Demographic and clinical characteristics

**Figure 1:**
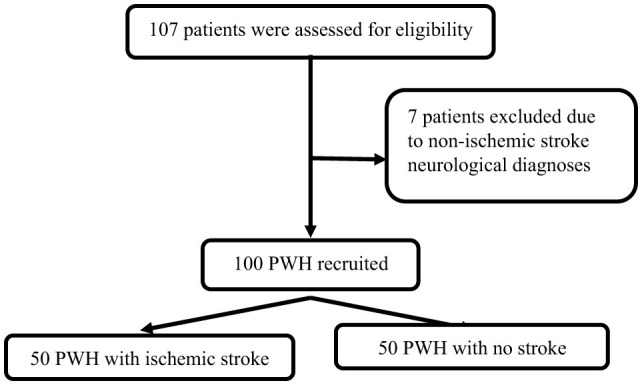
Overview of recruitment process.

In total, 50 cases and 50 controls were enrolled (Figure 1). For all cases, the stroke was their first ever stroke, and they presented with a median NIHSS score of 7 (3–17), and mRS score of 4 (2–5). The majority (*n* = 32; 64%) of cases had either large artery atherosclerosis or small artery occlusion. Compared to controls, cases were more likely to have hypertension, higher PWV, and increased cIMT. Cases had hyperlipidaemia, were more likely to use alcohol, had higher median diastolic blood pressure, and a higher WHO HIV clinical stage, but they were less likely to be on ART although these comparisons were not statistically significant. Similarly, cases had lower CD4 counts and higher HIV plasma viral loads compared to controls, but the difference was not statistically significant (Table 1).

**Table 1:** Comparison of baseline demographic and clinical characteristics between PWH with and without ischemic stroke.

**Characteristics**	**Cases (*n* = 50)**	**Controls (*n* = 50)**	***p*-Value**
Age, median (IQR)	42 (37–47)	42 (37–47)	0.766
Female, *n* (%)	33 (66)	33 (66)	0.999
Secondary school or higher, *n* (%)	38 (76)	42 (84)	0.371
Married/cohabiting, *n* (%)	20 (40)	27 (54)	0.161
Hypertension, *n* (%)	21 (42)	1 (2)	**0.001**
Diabetes mellitus, *n* (%)	2 (4)	0 (0)	0.152
Hyperlipidaemia, *n* (%)	5 (10)	0 (0)	0.022
Taking ART, *n* (%)	43 (86)	50 (100)	0.006
Family history of stroke, *n* (%)	13 (26)	8 (16)	0.220
Sudden death < 50 years in family, *n* (%)	5 (10)	3 (6)	0.461
Hypertension in family, *n* (%)	34 (68)	25 (50)	0.067
Diabetes mellitus in family, *n* (%)	10 (20)	14 (28)	0.349
Active smoker, *n* (%)	5 (10)	5 (10)	0.999
Passive smoker, *n* (%)	14 (28)	4 (8)	
Alcohol use, *n* (%)	18 (36)	7 (14)	0.011
**WHO clinical stage**, ***n*** **(%)**			0.030
Stage 1	35 (70)	44 (88)	
Stage 2	10 (20)	6 (12)	
Stage 3	5 (10)	0 (0)	
Stage 4	0 (0)	0 (0)	
NIHSS, median (IQR)	7 (3–17)	N/A	
mRS, median (IQR)	4 (2–5)	N/A	
TOAST classification, *n* (%)		N/A	
Large artery atherosclerosis	19 (38)		
Small artery occlusion	13 (26)		
Determined etiology	12 (24)		
Two or more causes	3 (6)		
Incomplete evaluation	3 (6)		
BMI (kg/m^2^), median (IQR)	25.6 (19.1–28.7)	24.4 (21–27.1)	0.856
Systolic BP (mmHg), median (IQR)	127 (111–150)	123 (113–135)	0.309
Diastolic BP (mmHg), median (IQR)	82 (74–93)	77 (70–89)	0.028
Total cholesterol (mmol/L), median (IQR)	4.55 (3.96–5.17)	4.29 (3.20–5.09)	0.250
Current CD4 (cells/μl), median (IQR)	259 (165–520)	452 (380–553)	0.030
HIV viral load (copies/ml), median (IQR)	0 (0–4,217)	0 (0–1,578)	0.007
HIV viral load suppressed *n* (%)	16/28 (57)	23/26 (88)	0.010
cIMT (mm), median (IQR)	0.79 (0.70–0.99)	0.63 (0.57–0.67)	**< 0.001**
cIMT ≥0.70, *n* (%)	38 (76)	14 (28)	**< 0.001**
PWV (m/s), median (IQR)	10.89 (9.99–12.15)	9.01 (7.98–9.67)	**< 0.001**
PWV ≥10.00 m/s, *n* (%)	37 (74)	0 (0)	**< 0.001**

Bolded text signifies *p* < 0.002.

ART, antiretroviral therapy; BMI, body mass index; cIMT, carotid intima-media thickness; mRS, modified Rankin scale; NIHSS, National Institute of Health stroke scale; PWV, pulse wave velocity; TOAST, Trial of ORG 10172 Acute Stroke Treatment; WHO, World Health Organization.

### 3.2 Inflammatory markers

**Figure 2:**
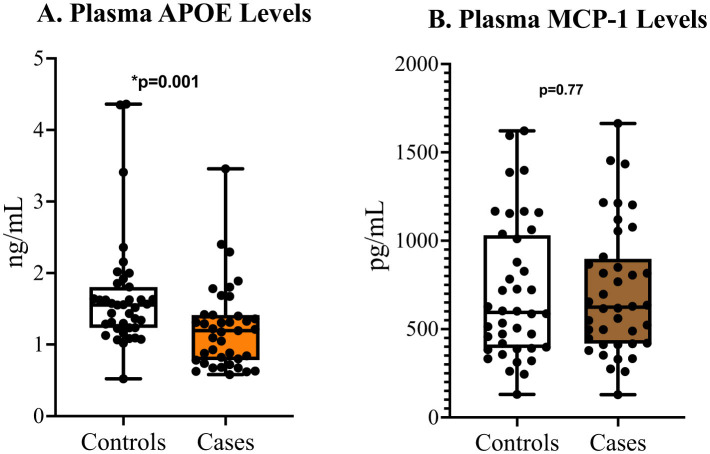
Comparison of plasma ApoE and MCP-1 levels between controls and cases.

Measured plasma ApoE was 1.20 (0.78–1.41) ng/ml among cases and 1.55 (1.23–1.81) ng/ml among controls. This difference was statistically significant (*p* = 0.001). The median level of MCP-1 was 622 (417–886) pg/ml among cases and 594 (394–1,024) pg/ml among controls (*p* = 0.772; Figure 2).

### 3.3 Independent predictors of ischemic stroke

After adjusting for potential confounders using a multivariable conditional logistic regression model, lower ApoE levels, abnormal cIMT ≥0.70 mm, and alcohol use were independent predictors of ischemic stroke (Table 2). The prediction model used had an area under the ROC curve of 0.84 (0.74–0.95), with a 95% confidence interval (Supplementary Figure S1).

**Table 2:** Multivariable conditional logistic regression analysis for factors associated with the probability of ischemic stroke in PWH.

**Variables**	**Adjusted OR (95% CI)**	***p*-Value (adj. OR)**
ApoE (ng/ml)	0.13 (0.03–0.68)	**0.015**
Alcohol use, *n* (%)	1,078 (4–267,933)	**0.013**
cIMT ≥0.70 mm	2.72 (1.08–6.85)	**0.033**
Diastolic BP (mmHg)	1.11 (0.99–1.25)	0.068

Bolded text signifies *p* < 0.05.

## 4 Discussion

Inflammatory markers have emerged as significant contributors to premature atherosclerosis and increased stroke risk, especially in PWH. Our data indicate that low ApoE plasma concentration is independently associated with young-onset HIV-associated non-cardioembolic ischemic stroke, but we did not find any association between MCP-1 plasma concentration and the latter. The association of ApoE genotype with stroke risk is well-established, but the relationship between circulating ApoE concentration and stroke has received little attention (Atadzhanov et al., 2013; Bennet et al., 2007).

Circulating ApoE concentration is associated with levels of major circulating lipids and lipoproteins, including total cholesterol, LDL, and HDL and triglycerides, but data on its association with stroke is conflicting (Vincent-Viry et al., 1998). Whereas, some studies have demonstrated an association between low ApoE plasma concentration with stroke (Mooijaart et al., 2006; van Vliet et al., 2007; Corsetti et al., 2012), others have not or have even demonstrated an opposite association, with higher circulating concentration of ApoE associated with a higher risk of incident stroke (Mooijaart et al., 2006). One meta-analysis of 9,587 individuals pulled from three different studies did not find any association between ApoE and stroke, although the study was in a largely HIV-uninfected population and cannot be generalized to PWH (Sofat et al., 2016). The presence of HIV and the potential impact of its interaction with ApoE makes PWH a special population which needs to be investigated on a large scale for this association.

Higher circulating levels of MCP-1 have been associated with increased long-term risk of stroke (Georgakis et al., 2019a). Conversely, low plasma MCP-1 levels have been associated with decreased risk for all-cause mortality and poor functional outcome after stroke (Xu et al., 2024). Given the well-established role of MCP-1 in premature atherosclerosis and stroke (Georgakis et al., 2019a,b), it is interesting that our study did not find any association. However, it is important to remember that our study was in young PWH and may have been limited by its small sample size. It is possible that these findings could be due to other factors such as biological variability or lack of sensitivity of this biomarker in our cohort. These findings also call for the interrogation of MCP-1 gene polymorphisms in our cohort. Nonetheless, our findings provide a foundation for further exploration especially in sub-Saharan Africa where the burden of HIV-associated stroke is highest.

Our data also show that alcohol use and abnormal cIMT are each independently associated with non-cardioembolic ischemic stroke among young PWH. Several studies have shown that alcohol use is an important risk factor for stroke with an inverse relationship depending on quantities consumed, with some studies suggesting that alcohol use in moderation is actually protective (Smyth et al., 2023; Reynolds et al., 2003; Zhang et al., 2014). These studies have been in HIV-uninfected individuals, but alcohol use like abnormal cIMT is associated with atherosclerosis (Kiechl et al., 1998; Da Luz and Coimbra, 2001; Tolstrup, 2007). Our findings ultimately place emphasis on the fact that the actual mechanism of stroke in this population of young-onset HIV-associated ischemic stroke is likely multifactorial, with the foundation on which stroke occurs likely to be premature atherosclerosis as evidenced by the abnormal cIMT and alcohol use being independent predictors (Kim and Kim, 2019; Flint et al., 2019).

Our study has several limitations. Some findings were based on information from the routine care available at the hospital during the study. Resource limitations also meant that not every participant was thoroughly evaluated for stroke risk factors such as hyperlipidaemia and diabetes (Supplementary Table S1). Failure to do brain imaging rendered it impossible for some patients to be included in the study, and this was often related to death or discharge prior to enrollment. This introduces the possibility of selection bias, which could have systematically excluded the most severely ill subjects and/or subjects with late-stage HIV. We neither recruited healthy participants nor thoroughly evaluated the ART history/regimen our participants were on, but first line therapy was dolutegravir based. However, a strength of this study is that all participants received a thorough clinical evaluation by trained neurologists, making the clinical characterizations of this cohort more reliable than in many other sub-Saharan African populations in which participants are assessed by non-neurologist healthcare workers.

This was a single-center study with a small sample size of Zambian patients at a national referral hospital, which might make the generalizability of our findings to other populations limited. The results should be interpreted with caution and need to be verified by further research, including correlating these results with individuals' ApoE and MCP-1 gene polymorphisms. These results are in the setting of an observational study in which cut-off points for both markers with adequate clinical sensitivity and specificity have not yet been validated. Nevertheless, our expectation is that these results are likely generalizable to similar settings in other populations of PWH in SSA, and they provide preliminary data that can be used to guide the development of interventions to reduce stroke risk in young PWH.

In summary, we observed that low plasma ApoE levels are associated with young-onset HIV-associated non-cardioembolic ischemic stroke. Low plasma ApoE, abnormal cIMT, and alcohol use were each independently associated with stroke. Although these findings should be taken with caution due to limited sample size, they call attention to the multifactorial nature of stroke risk factors in young PWH. A comprehensive approach addressing all of these risk factors is needed to effectively reduce the stroke burden in this population. Additional studies with larger sample sizes are needed to further explore the association of these inflammatory markers, including their individual gene polymorphisms with young-onset HIV-associated stroke.

## Data Availability

The original contributions presented in the study are included in the article/Supplementary material, further inquiries can be directed to the corresponding author.

## Footnotes

1 In **Sections 2.5** and **2.6**, the initials given are those of the relevant author. MC: Mike Chisha; SZ: Stanley Zimba; MA: Masharip Atadzhanov; DS: Deanna Saylor.

## References

[B1] Alberti K. G. M. M. Zimmet P. Z. (1998). Definition, diagnosis and classification of diabetes mellitus and its complications. Part 1: diagnosis and classification of diabetes mellitus Provisional report of a WHO consultation. Diabet. Med. 15, 539–553. doi: 10.1002/(SICI)1096-9136(199807)15:7<539::AID-DIA668>3.0.CO;2-S 9686693

[B2] Albuquerque V. M. Zírpoli J. C. de Barros Miranda-Filho D. de Albuquerque M. F. Montarroyos U. R. de Alencar Ximenes R. A. . (2013). Risk factors for subclinical atherosclerosis in HIV-infected patients under and over 40 years: a case–control study. BMC Infect. Dis. 13, 1–13. doi: 10.1186/1471-2334-13-274 23773229 PMC3686657

[B3] Alonso-Villaverde C. Coll B. Parra S. Montero M. Calvo N. Tous M. . (2004). Atherosclerosis in patients infected with HIV is influenced by a mutant monocyte chemoattractant protein-1 allele. Circulation 110, 2204–2209. doi: 10.1161/01.CIR.0000143835.95029.7D 15466648

[B4] Atadzhanov M. Mwaba M. H. Mukomena P. N. Lakhi S. Rayaprolu S. Ross O. A. . (2013). Association of the APOE, MTHFR and ACE genes polymorphisms and stroke in Zambian patients. Neurol. Int. 5:e20. doi: 10.4081/ni.2013.e20 24416484 PMC3883065

[B5] Bennet A. M. Di Angelantonio E. Ye Z. Wensley F. Dahlin A. Ahlbom A. . (2007). Association of apolipoprotein E genotypes with lipid levels and coronary risk. JAMA 298, 1300–1311. doi: 10.1001/jama.298.11.1300 17878422

[B6] Charo I. F. Taubman M. B. (2004). Chemokines in the pathogenesis of vascular disease. Circ. Res. 95, 858–866. doi: 10.1161/01.RES.0000146672.10582.17 15514167

[B7] Coll B. Parra S. Alonso-Villaverde C. Aragonés G. Montero M. Camps J. . (2007). The role of immunity and inflammation in the progression of atherosclerosis in patients with HIV infection. Stroke 38, 2477–2484. doi: 10.1161/STROKEAHA.106.479030 17673719

[B8] Corsetti J. P. Gansevoort R. T. Bakker S. J. Navis G. Sparks C.E. Dullaart R.P. (2012). Apolipoprotein E predicts incident cardiovascular disease risk in women but not in men with concurrently high levels of high-density lipoprotein cholesterol and C-reactive protein. Metabolism 61, 996–1002. doi: 10.1016/j.metabol.2011.11.010 22225956

[B9] Da Luz P. L. Coimbra S. R. (2001). Alcohol and atherosclerosis. An. Acad. Bras. Ciênc. 73, 51–55. doi: 10.1590/S0001-37652001000100006 11246269

[B10] Flint A. C. Conell C. Ren X. Banki N. M. Chan S. L. Rao V. A. . (2019). Effect of systolic and diastolic blood pressure on cardiovascular outcomes. N. Engl. J. Med. 381, 243–251. doi: 10.1056/NEJMoa1803180 31314968

[B11] Georgakis M. K. Gill D. Rannikmäe K. Traylor M. Anderson C. D. Lee J. M. . (2019a). Genetically determined levels of circulating cytokines and risk of stroke: role of monocyte chemoattractant protein-1. Circulation 139:256–268. doi: 10.1161/CIRCULATIONAHA.118.035905 30586705 PMC7477819

[B12] Georgakis M. K. Malik R. Björkbacka H. Pana T. A. Demissie S. Ayers C. . (2019b). Circulating monocyte chemoattractant protein-1 and risk of stroke: meta-analysis of population-based studies involving 17 180 individuals. Circ. Res. 125, 773–782. doi: 10.1161/CIRCRESAHA.119.315380 31476962 PMC6763364

[B13] Goldstein L. B. Jones M. R. Matchar D. B. Edwards L. J. Hoff J. Chilukuri V. . (2001). Improving the reliability of stroke subgroup classification using the Trial of ORG 10172 in Acute Stroke Treatment (TOAST) criteria. Stroke 32, 1091–1097. doi: 10.1161/01.STR.32.5.1091 11340215

[B14] Gu L. Rutledge B. Fiorillo J. Ernst C. Grewal I. Flavell R. . (1997). *In vivo* properties of monocyte chemoattractant protein-1. J. Leukoc. Biol. 62, 577–580. doi: 10.1002/jlb.62.5.577 9365111

[B15] Harris P. A. Taylor R. Minor B. L. Elliott V. Fernandez M. O'Neal L. . (2019). The REDCap consortium: building an international community of software platform partners. J. Biomed. Inform. 95:103208. doi: 10.1016/j.jbi.2019.103208 31078660 PMC7254481

[B16] Harris P. A. Taylor R. Thielke R. Payne J. Gonzalez N. Conde J. G. (2009). Research electronic data capture (REDCap)—a metadata-driven methodology and workflow process for providing translational research informatics support. J. Biomed. Inform. 42, 377–381. doi: 10.1016/j.jbi.2008.08.010 18929686 PMC2700030

[B17] Hsue P. Y. Deeks S. G. Hunt P. W. (2012). Immunologic basis of cardiovascular disease in HIV-infected adults. J. Infect. Dis. 205, S375–82. doi: 10.1093/infdis/jis200 22577211 PMC3349295

[B18] Ibáñez L. Velli P. S. Font R. Jaén A. Royo J. Irigoyen D. . (2014). HIV-infection, atherosclerosis and the inflammatory pathway: candidate gene study in a Spanish HIV-infected population. PLoS ONE 9:e112279. doi: 10.1371/journal.pone.0112279 25383745 PMC4226484

[B19] Kiechl S. Willeit J. Rungger G. Egger G. Oberhollenzer F. Bonora E. (1998). Alcohol consumption and atherosclerosis: what is the relation? Prospective results from the Bruneck Study. Stroke 29, 900–907. doi: 10.1161/01.STR.29.5.900 9596232

[B20] Kim H.-L. Kim S.-H. (2019). Pulse wave velocity in atherosclerosis. Front. Cardiovasc. Med. 6:41. doi: 10.3389/fcvm.2019.00041 31024934 PMC6465321

[B21] Lorenz M. W. Markus H. S. Bots M. L. Rosvall M. Sitzer M. (2007). Prediction of clinical cardiovascular events with carotid intima-media thickness: a systematic review and meta-analysis. Circulation 115, 459–467. doi: 10.1161/CIRCULATIONAHA.106.628875 17242284

[B22] Lyden P. Brott T. Tilley B. Welch K. M. Mascha E. J. Levine S. . (1994). Improved reliability of the NIH Stroke Scale using video training. NINDS TPA Stroke Study Group. Stroke 25, 2220–2226. doi: 10.1161/01.STR.25.11.2220 7974549

[B23] Mooijaart S. P. Berbée J. F. van Heemst D. Havekes L. M. de Craen A. J. Slagboom P. E. . (2006). ApoE plasma levels and risk of cardiovascular mortality in old age. PLoS Med. 3:e176. doi: 10.1371/journal.pmed.0030176 16671834 PMC1457005

[B24] Mulenga S. F. (2024). Population growth as a cause of human development in Zambia. Sustain. Dev. 7, 89–110. doi: 10.52589/AJESD-6O65HARG

[B25] Nelson R. H. (2012). Hyperlipidemia as a risk factor for cardiovascular disease. Prim. Care. 40:195. doi: 10.1016/j.pop.2012.11.003 23402469 PMC3572442

[B26] Norton G. R. Majane O. H. Maseko M. J. Libhaber C. Redelinghuys M. Kruger D. . (2012). Brachial blood pressure–independent relations between radial late systolic shoulder-derived aortic pressures and target organ changes. Hypertension 59, 885–892. doi: 10.1161/HYPERTENSIONAHA.111.187062 22331378

[B27] Piconi S. Clerici M. (2013). HIV replication, inflammation and atherogenesis: dangerous liaisons. AIDS 27, 1521–1522. doi: 10.1097/QAD.0b013e32835fffb2 23945506

[B28] Piconi S. Parisotto S. Rizzardini G. Passerini S. Meraviglia P. Schiavini M. . (2013). Atherosclerosis is associated with multiple pathogenic mechanisms in HIV-infected antiretroviral-naive or treated individuals. AIDS 27, 381–389. doi: 10.1097/QAD.0b013e32835abcc9 23079800

[B29] Reynolds K. Lewis B. Nolen J. D. L. Kinney G. L. Sathya B. He J. (2003). Alcohol consumption and risk of stroke: a meta-analysis. JAMA 289, 579–588. doi: 10.1001/jama.289.5.579 12578491

[B30] Rovin B. H. Lu L. Saxena R. (1999). A novel polymorphism in the MCP-1 gene regulatory region that influences MCP-1 expression. Biochem. Biophys. Res. Commun. 259, 344–348. doi: 10.1006/bbrc.1999.0796 10362511

[B31] Roy H. Bhardwaj S. Yla-Herttuala S. (2009). Molecular genetics of atherosclerosis. Hum Genet. 125, 467–491. doi: 10.1007/s00439-009-0654-5 19301036

[B32] Scale S. (2008). Modified Rankin Scale. Available online at: http://www/strokecenterorg/trials/scales/rankinhtml (Accessed March 1, 2022).

[B33] Schaefer E. J. Gregg R. E. Ghiselli G. Forte T. M. Ordovas J. M. Zech L. A. . (1986). Familial apolipoprotein E deficiency. J. Clin. Invest. 78, 1206–1219. doi: 10.1172/JCI112704 3771793 PMC423806

[B34] Smyth A. O'Donnell M. Rangarajan S. Hankey G. J. Oveisgharan S. Canavan M. . (2023). Alcohol intake as a risk factor for acute stroke: the INTERSTROKE study. Neurology 100, e142–e153. doi: 10.1212/WNL.0000000000201388 36220600 PMC9841450

[B35] Sofat R. Cooper J. A. Kumari M. Casas J. P. Mitchell J. P. Acharya J. . (2016). Circulating apolipoprotein E concentration and cardiovascular disease risk: meta-analysis of results from three studies. PLoS Med. 13:e1002146. doi: 10.1371/journal.pmed.1002146 27755538 PMC5068709

[B36] Tolstrup J Grønbaek M. (2007). Alcohol and atherosclerosis: recent insights. Curr. Atheroscler. Rep. 9, 116–124. doi: 10.1007/s11883-007-0007-6 17877920

[B37] Touboul P. J. Hennerici M. G. Meairs S. Adams H. Amarenco P. Bornstein N. . (2012). Mannheim carotid intima-media thickness and Plaque consensus (2004–2006–2011) an update on behalf of the advisory board of the 3rd, 4th and 5th watching the risk symposia, at the 13th, 15th and 20th European Stroke conferences, Mannheim, Germany, 2004, Brussels, Belgium, 2006, and Hamburg, Germany, 2011. Cerebrovasc. Dis. 34, 290–296. doi: 10.1159/000343145 23128470 PMC3760791

[B38] Triant V. A. (2012). HIV: infection and coronary heart disease: an intersection of epidemics. J. Infect. Dis. 205, S355–S361. doi: 10.1093/infdis/jis195 22577208 PMC3349293

[B39] Triant V. A. Lee H. Hadigan C. Grinspoon S. K. (2007). Increased acute myocardial infarction rates and cardiovascular risk factors among patients with human immunodeficiency virus disease. J. Clin. Endocrinol. Metab. 92, 2506–2512. doi: 10.1210/jc.2006-2190 17456578 PMC2763385

[B40] van Vliet P. Mooijaart S. P. De Craen A. J. Rensen P. C. van Heemst D. Westendorp R. G. (2007). Plasma levels of apolipoprotein E and risk of stroke in old age. Ann. N. Y. Acad. Sci. 1100, 140–147. doi: 10.1196/annals.1395.012 17460172

[B41] Vincent-Viry M. Schiele F. Gueguen R. Bohnet K. Visvikis S. Siest G. (1998). Biological variations and genetic reference values for apolipoprotein E serum concentrations: results from the STANISLAS cohort study. Clin. Chem. 44, 957–965. doi: 10.1093/clinchem/44.5.957 9590368

[B42] Whitworth J. (2003). World Health Organization, International Society of Hypertension Writing Group. J. Hypertens. 21, 1983–1992. doi: 10.1097/00004872-200311000-00002 14597836

[B43] Xu Q. Liu Y. Tian X. Xia X. Zhang Y. Zhang X. . (2024). Monocyte chemoattractant protein-1, inflammatory biomarkers, and prognosis of patients with ischemic stroke or transient ischemic attack: fndings from a Nationwide Registry Study. J. Am. Heart Assoc. 13:e035820. doi: 10.1161/JAHA.124.035820 39119971 PMC11963953

[B44] Zhang C. Qin Y. Y. Chen Q. Jiang H. Chen X. Z. Xu C. L. . (2014). Alcohol intake and risk of stroke: a dose–response meta-analysis of prospective studies. Int. J. Cardiol. 174, 669–677. doi: 10.1016/j.ijcard.2014.04.225 24820756

[B45] Zimba S. Nutakki A. Chishimba L. Chomb M. Bahouth M. Gottesman R. F. . (2021). Risk factors and outcomes of HIV-associated stroke in Zambia. AIDS 35, 2149–2155. doi: 10.1097/QAD.0000000000002999 34138769

