# Supplementary material for: Plasma apolipoprotein E and monocyte chemoattractant protein-1 levels in young people with HIV and ischemic stroke in Lusaka, Zambia
Source: Front Stroke. 2025 Nov 20;4:1595809. doi: 10.3389/fstro.2025.1595809 (PMC12802788; doi:10.3389/fstro.2025.1595809)
Supplement: Supplementary file 2 [file Table_2.doc]

**Supplemental Table 1.** Proportion of participants with results from laboratory, imaging and other ancillary investigations available

| **Investigation** | **Proportion of Participants with Results n (%)** |
| --- | --- |
| Lipid panel | 56 (56%) |
| Hypercoagulable workup | 26 (26%) |
| CT brain | 47 (47%) |
| MRI brain | 8 (8%) |
| Carotid ultrasound | 100 (100%) |
| CT or MR angiogram | 5 (5%) |
| Electrocardiogram | 69 (69%) |
| Echocardiogram | 86 (57%) |
| CD4 count | 89 (89%) |
| Viral load | 88 (88%) |

**Supplemental Figure 1.** Area under ROC curve analysis for Ischaemic stroke predictive model
